# Dorsolateral medullary infarction registry: a study protocol for a prospective, multicentric registry

**DOI:** 10.1186/s12883-020-02030-6

**Published:** 2021-01-12

**Authors:** Jing Wang, Pei Liu, Zhongzhong Liu, Ye Tian, Guilian Zhang, Jun Guo, Li Li, Zhiqin Liu, Zucheng Han, Wenjuan Lin, Xuemei Lin, Qingli Lu, Yan Liu, Qiaoqiao Chang, Songdi Wu

**Affiliations:** 1 Department of Neurology, First Hospital of Xi’an, 710002 Xi’an, Shaanxi Province China; 2 Department of Neurology, the First Affiliated Hospital of Northwestern University, 710002 Xi’an, Shaanxi Province China; 3 Department of Neurology, Third Hospital of Xi’an, the Affiliated Hospital of Northwest University, 710018 Xi’an, Shaanxi Province China; 4 Department of Neurology, the Second Affiliated Hospital of Xi’an Jiaotong University, 710004 Xi’an, Shaanxi Province China; 5 Department of Neurology, Tangdu Hospital, Air Force Military Medical University, 710038 Xi’an, Shaanxi Province China; 6 Department of Neurology, Xijing Hospital, Air Force Military Medical University, 710032 Xi’an, Shaanxi Province China; 7 Department of Neurology, Xi’an Central Hospital, Xi’an Jiaotong University School of Medicine, 710003 Xi’an, Shaanxi Province China; 8 Encephalopathy Hospital, Shanxi Provincial Hospital of Traditional Chinese Medicine, 710077 Xi’an, Shaanxi Province China; 9 College of Life Sciences, Northwest University, 710069 Xi’an, Shaanxi Province China

**Keywords:** Dorsolateral medullary infarction, Neurotrophic keratopathy, Corneal innervation, Ocular surface environment

## Abstract

**Background:**

Dorsolateral medullary infarction is a typical cerebral infarction which is characterized by Wallenberg’s syndrome. Neurotrophic keratopathy is an uncommon consequence of dorsolateral medullary infarction. At present, the protocol is aimed to study the dynamic changes in corneal innervation and the ocular surface environment after dorsolateral medullary infarction.

**Methods:**

This study will involve consecutive data from all medical records of patients within 7 days of acute dorsolateral medullary infarction onset at the Departments of Neurology from 10 collaborating stroke centers. Eligible patients will mainly be characterized based on detailed physical examinations, multimodal imaging, and corneal related examinations and patients will be followed-up for 2 years. Neurotrophic keratopathy after dorsolateral medullary infarction is the primary endpoint. The dynamic histological corneal innervation and ocular surface environment after dorsolateral medullary infarction will be observed during the follow-up period.

**Discussion:**

This multicentric, prospective registry is the first to identify and characterize the dynamic changes of corneal innervation and the ocular surface environment after acute dorsolateral medullary infarction. The significance of the study is to emphasize that the curative effect is based on the doctors’ identification of the disease in the earliest stage before irreversible damage occurs to the cornea.

**Trial registration:**

The registry was registered (ChiCTR-OPC-17,011,625) on June 11, 2017.

## Background

Dorsolateral medullary infarction (DLMI) is diagnosed based on characteristic clinical symptoms of Wallenberg’s syndrome with imaging assistance. The most common symptoms are sudden vertigo and vomiting; Horner’s syndrome; gait ataxia and a recognizable tendency to fall sideways; dysphagia, hoarseness and intractable hiccups; and ipsilateral facial hypalgesia, thermohypesthesia, and contralateral impairment of pain and temperature sensation over the trunk and limbs. Neurotrophic keratopathy (NK) is an uncommon delayed-onset complication of DLMI [1, 2].

NK develops when corneal nerves are damaged [3]. To the best of our knowledge, most corneal nerves originate from the ophthalmic division of the trigeminal nerve, distending bilaterally from the lowest part of the spinal trigeminal nucleus. The lowest part of the spinal trigeminal nucleus includes second-order sensory cell bodies of polymodal nociceptor neurons, cold thermoreceptor neurons, and selective mechano-nociceptor neurons. Therefore, DLMI lesions (especially at the lowest part of the spinal nuclei) could cause infrequent ophthalmic manifestations, including reduced corneal sensation, dry eye, foreign body sensation, and corneal ulceration [2].

In recent years, a few reports have indicated that ulcerative keratonosus can occur secondary to DLMI [1, 2, 4, 5]. Hipps et al. reported a case of neurotrophic corneal ulceration and hypopyon after DLMI and indicated that it is important to prevent NK after DLMI [1]. Cidad et al. described the cases of three patients who experienced persistent epithelial defects for 1–7 years after Wallenberg syndrome, emphasized the importance in early detection of corneal erosions, persistent epithelial ulcerations, and hypopyon after DLMI onset [4]. We previously reported the case of a patient with NK after DLMI who had a relatively successful treatment outcome [2]. Pellegrini et al. also reported the case of a 47-year-old man with NK after 1 month of having Wallenberg syndrome [5]. We have observed many similar cases in our clinical practice; in these cases, the patients’ conditions were difficult to manage, and the curative effect and prognosis were not ideal. Therefore, we designed a multicenter, prospective registry to observe the dynamic changes in clinical corneal manifestation after DLMI.

Various theories have been proposed to explain the pathogenesis of NK [3, 6–10]. Corneal denervation could lead to impairment of sensory and trophic function. Ferrari et al. found an increase in cellular apoptosis and a reduction in proliferation, which suggests that damaged tropism support of corneal nerves leads to corneal erosion and ulceration [11]. A dry corneal surface due to diminished lacrimal secretions and damaged ocular homeostasis could destroy the metabolic loop of providing nutrition and removing cell waste, and ultimately result in corneal ulceration [3].

At present, the dynamic changes in corneal innervation and the ocular surface environment after DLMI are unclear. From a clinical perspective, the DLMI registry is designed to (1) monitor dynamic changes in corneal innervation and in the ocular surface environment after DLMI, (2) identify individuals with NK after DLMI, and (3) to assist with timely intervention.

## Methods

### Study design

**Fig. 1:**
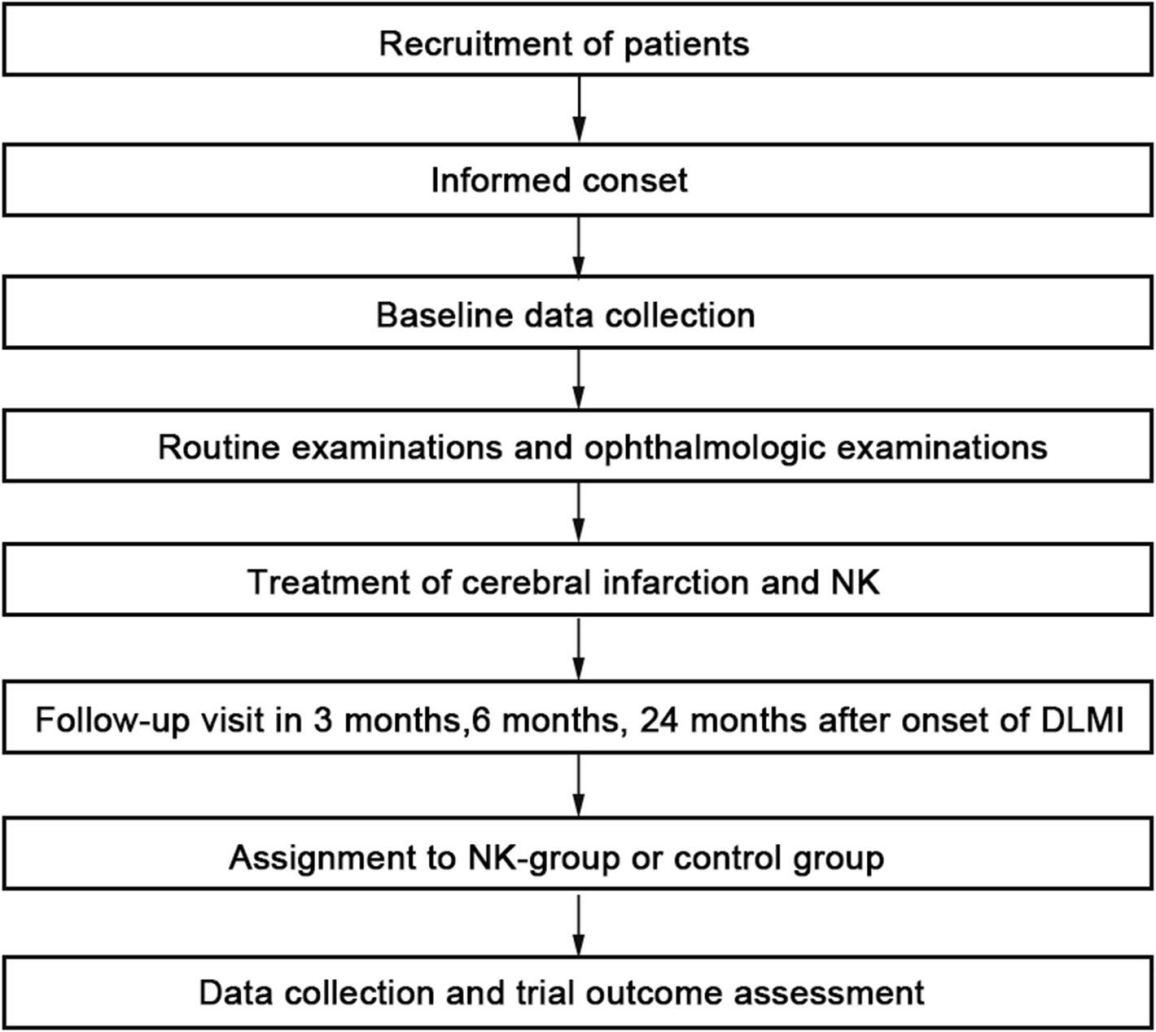
study design

The Dorsolateral Medullary Infarction registry is a multicentric, prospective registry in Xi’an (Fig. 1). The Ethics Committee of the First Hospital of Xi’an and all other participating centers have approved the protocol. Each eligible participant with DLMI has provided written informed consent. For each enrolled patient, their personal information will be recorded and kept in a secure folder. To protect their privacy, the patients’ personal information will only be made accessible to the researchers. The registry (no. ChiCTR-OPC-17,011,625) was registered on June 11, 2017; the study was designed in accordance with the Declaration of Helsinki.

### Recruitment

 The study recruits all patients diagnosed with DLMI from the Neurology Departments of 10 collaborating stroke centers in different regions. Consecutive recruitment of patients commenced in December 2019 and will end by December 2021. For achieving adequate participant enrolment, eligible patients will be recruited via public mediums, such as social media, WeChat moments and subscriptions, posters on the walls of hospital health clinics, speaking with patients and their families at the Department of Neurology, and via word of mouth at neurology outpatient clinics at each hospital.

### Participant screening

This registry enrolls patients with acute DLMI who meet the inclusion (but not the exclusion) criteria. Each hospital will designate two to three neurologists for patient recruitment, examination supervision, and treatment. Any acute ischemic stroke (AIS) patient with DLMI who meets the following criteria is eligible:

Inclusion criteria:

1. Age ≥ 18 years;
2.  Acute DLMI with typical clinical and/or imaging manifestations;
3.  Onset to registry (OTR) time < 7 days;
4.  Providing informed consent;
5.  Willing to participate in a follow-up visit.

Exclusion criteria:

1. No symptoms or signs of cerebral infarction;
2. Prior corneal disease;
3. Refusal to provide informed consent.

Exit/termination criteria:

1. Lost to follow-up;
2. Not following the research schedule, including a lack of compliance (medication, examinations, and interview plan), adverse events (such as severe cognitive impairment and death), and other events that discourage patients from cooperating with the study schedule.

### Measurement

#### Scores for clinical evaluation

When DLMI occurs, demographic and general information, including smoking status, blood pressure, and blood sugar and blood lipid levels will be collected. Characteristic factors that affect the prognosis will also be analyzed, including the National Institute of Health Stroke Scale (NIHSS), modified Rankin scale (mRS), infarction volume, infarction position, and compliance (number of days on antiplatelet or anticoagulation drugs, statins).

#### Systemic examinations

Any AIS patient suspected of having DLMI should undergo comprehensive systemic examinations and finish the examinations (Table 1), including: brain magnetic resonance imaging (MRI), cardiac examinations (general 12-lead electrocardiogram, 24-h electrocardiogram, transthoracic echocardiography), and vascular evaluation; at least one of the following examinations is available: cervical vascular ultrasound, computed tomography angiography and/or digital subtraction angiography of the head and neck vessels, and magnetic resonance angiography of the intracranial vessels.

**Table 1:** Overview of the study assessment

Assessment	Domain	Baseline evaluation (T0)	3-month evaluation (T1)	6-month symptoms (T2)	12-month evaluation (T3)	24-month evaluation (T4)
**Interview** • Demographic registry	General condition	√	√	√	√	√
• Past disease history		√	√	√	√	√
• Vascular risk factors (e.g., smoking, alcohol consumption)		√	√	√	√	√
• Family history		√	**-**	**-**	**-**	**-**
**Clinical assessment** • Blood pressure, instant blood sugar	Health condition	√	√	√	√	√
• National Institutes of Health Stroke Scale, Modified Rankin Scale		√	√	√	√	√
• Physical and neurological examination		√	√	**-**	√	√
**Primary outcome measure**	Corneal function	√	√	**-**	√	√
• OSDI	Tear secretion	√	√	-	√	√
• Schirmer test		√	√	-	√	√
• Non-invasive tear film breakup time		√	√	-	√	√
• Thickness of lipid layer of lacrimal film		√	√	-	√	√
• PBR		√	√	-	√	√
• Corneal sensation	Corneal sensation	√	√	-	√	√
• BSCVA	Vision	√	√	-	√	√
• ASOCT	Anterior segment	√	√	-	√	√
• Meibomian gland and lipid layer		√	√	-	√	√

OSDI: Ocular Surface Disease Index; BSCVA: best-spectacles corrected visual acuity; ASOCT: anterior segment optical coherence tomography; IVCM: in vivo confocal microscopy; PBR: partial blinking rate

#### Ophthalmologic examinations

Corneal innervation and the ocular surface environment are important in the development and prognosis of neurotrophic keratitis. The enrolled patients will undergo ophthalmologic examinations of corneal function within 7 days of DLMI onset. The ophthalmologic examinations of corneal function include the ocular surface disease index (OSDI), best-spectacles corrected visual acuity (BSCVA), corneal sensation, anterior segment optical coherence tomography (ASOCT), in vivo confocal microscopy (IVCM), Schirmer test, non-invasive tear film breakup time, analysis of the meibomian gland and lipid layer, thickness of the lipid layer of the lacrimal film, and the partial blinking rate (PBR).

The OSDI is a very important index to score the degree of lacrimal secretion in the eligibility-confirmation step [12]. A higher score indicates a more serious condition. The BSCVA indicates the condition of visual impairment. Clinically, corneal sensation is assessed using a “wisp” of cotton applied to both corneas; the number of times the patient’s affected eye blinks is noted and is compared with their other eye. NK patients always show reduced blinking. ASOCT allows for the measurement and visualization of microscopic structures as well as evaluation of corneal pathologic features; this method also allows for dynamic analysis in the follow-up period [13–16]. IVCM can be used to evaluate living human cornea both in healthy and pathological conditions [17, 18]. Lacrimal secretion can be assessed using the Schirmer test, non-invasive tear film breakup time, tear meniscus height, and the PBR [3, 19].

#### Assessment and outcome evaluation

A prerequisite for the diagnosis of NK is corneal defect. The corneal condition is defined according to the severity rating proposed by Dua et al. [3]. We will quantify the severity rating of the corneal function based on a number of factors on a scale from 0 to 3 (normal to severe).

- Normal (score = 0): no epithelial or stromal defect.
- Mild (epithelial changes without epithelial defect) (score = 1): epithelial irregularity without a frank epithelial defect; tear film instability and symptoms with reduced or absent sensations in one or more quadrants of the cornea.
- Moderate (epithelial defect without stromal defect) (score = 2): frank persistent epithelial defect and corneal hypoesthesia/anesthesia.
- Severe (stromal involvement) (score = 3): stromal involvement from the corneal ulcer to lysis to perforation, with corneal hypoesthesia/an aesthesia.

Based on the results, enrolled patients will be divided into NK and control groups for further analysis.

### Treatment

For treatment, we will administer clopidogrel, aspirin, and statin drugs to all registered patients according to the guidelines for acute cerebral infarction (clopidogrel, aspirin, and statin drugs within 21 days after infarction onset, followed by clopidogrel or aspirin and statin drugs later on) [20]. Meanwhile, we will ask patients to control hazard factors, such as hypertension, hyperglycemia, hyperlipemia, hyperhomocysteinemia, and obesity.

Due to a high incidence of cardiopulmonary arrest, the importance of managing DLMI will be kept in mind by each clinician and nurse as early as the first reception [20, 21]. Patients who are diagnosed with DLMI will be admitted to the intensive care unit, and the relatives of patients will be informed of the patient’s condition. While treating cerebral infarction, we will pay attention to the prophylaxis of the post-stroke complications, including administering low-molecular weight heparin for deep vein thrombosis, enteral nutrition for consumption and misinhalation, gastric mucosa protection for Cushing ulcer, antibiotic treatment for ventilator-associated pneumonia, and catheter-associated urinary tract infection, among others. If the condition permits, related examinations will be performed early on [20, 21].

The treatment of NK will be divided into three aspects, including medical management, non-surgical intervention, and surgical management. Through osmosis, nutrients and metabolites move in and out of the cornea; thus, it is suitable for clinicians to use local drugs for substituting tears, thereby providing binding sites for growth factors to promote healing or to combat inflammatory. When an infection is suspected, empirical treatment with antibiotics will be advised. Researchers have stated that the upper bulbar conjunctiva appearing white is a useful early manifestation of the toxic effect of antibiotics in the “up-down” test (e.g., quinolones and aminoglycosides) [22]. Tarsorrhaphy or botulinum toxin injection will be used as non-surgical interventions to protect against environmental infections and to avoid aggravating the condition [23, 24]. If cornea perforation occurs, an amniotic membrane graft or corneal graft will be chosen [3].

### Follow-up visit

Corneal innervation and the ocular surface environment will be examined 3, 6, 12, and 24 months after the onset of DLMI (the schedule of assessments is depicted in Table 1). Face-to-face visits will occur at 3, 12, and 24 months, whereas a telephone visit will occur at 6 months. Corneal function and ocular comorbidities will be reevaluated in a timely manner so as to observe the effect and adjust the treatment program. Previous research did not find a direct relationship between the severity of NK and visual recovery or outcome [6]. Visual impairment of NK would be the most common cause for patients to see a doctor. It is always neglected because diminished lacrimal secretions may not be obvious. Clinicians should pay attention to lacrimal secretion after DLMI.

### Outcome

#### Primary outcome

In this registry, NK is the primary endpoint. For outcome assessment, researchers will be specifically trained and will be familiar with each examination. Outcomes will be assessed using a consistent grading standard.

Corneal innervation and the ocular surface environment will be evaluated at baseline and at 3, 6, 12, and 24 months after the onset of DLMI. Cellular microscopic changes in the corneal epithelium and stroma will be examined using in vivo confocal microscopy, and the grade of corneal defect will be based on the currently established grading system [3].

Meanwhile, the manifestations of NK are a combination of diminished lacrimal secretions, diminished protective blink reflexes, photophobia, or impaired vision [3, 25, 26]. The following examination results would support a diagnosis of NK: high OSDI score, poor BSCVA, weak corneal sensation, damaged anterior segment, meibomian gland, lipid layer, and lipid layer of the lacrimal film, high Schirmer test score, shortened non-invasive tear film breakup time, and a reduced PBR. Differences will be analyzed between the NK and control groups as well as between bilateral eyes.

#### Sample size

This cohort study is intended to observe and monitor the dynamic changes in corneal innervation and in the ocular surface environment after acute DLMI. Because NK after DLMI is only reported in individual cases, no research has provided enough information to accurately calculate the necessary sample size for the present study. To the best of our knowledge, this study is the first registration study of DLMI so far. To determine the sample size, we reviewed the hospital admission rates of the 10 collaborating centers and asked 10 neurologic and three statistical experts for advice on the retrospective analysis of the clinical data. After several meetings, we agreed that a sample size of > 80 patients should be sufficient to gather enough information, except for critically-ill patients who need to be in the intensive care unit.

#### Timeline

Consecutive recruitment of patients commenced in December 2019 and will end by December 2021. All DLMI patients will undergo examinations and treatment as well as 2 years of follow-up.

#### Data collection and management

To ensure the accuracy of outcome assessment, researchers will be specifically trained and will be well informed about each examination procedure. A unified data collection system created by the Department of Neurology of the First Hospital of Xi’an (Shaanxi Province, China) will be used by each researcher in the member hospitals. The data collector will use an electronic case report form (eCRF) to record the original data and will perform an additional check for the accuracy of the entry. Once the information is confirmed, the eCRF of each patient will be saved in a portable document format. The data collectors will then send the data through the website to the Data Monitoring Committee.

#### Data monitoring and auditing

The Data Monitoring Committee comprises three members, including a neurologist, an ophthalmologist, and a statistician. In combination with data on clinical symptoms and examination results, the neurologist and ophthalmologist will decide whether NK (the primary endpoint) has occurred. The coordinating center has created a WeChat work group dedicated to communicating about work issues. There is also a WeChat group used to contact patients regarding their recent physical condition, to remind them of regular return visits, and to answer questions from patients. The Data Monitoring Committee will input all types of data in a timely manner (once a quarter), process data regularly, and report the results to superiors. When data collectors encounter adverse events and safety issues, these will be reported to the main researchers and Data Monitoring Committee within 24 h. The independent safety monitoring authority will make the final decision regarding study termination.

#### Statistical analyses

We will compute the descriptive statistics for the general demographic characteristics for the overall study samples. Clinically relevant variables, including general factors (smoking status, blood pressure, and blood sugar and blood lipid levels) and characteristic factors (NIHSS, mRS, infarction volume, infarction position, and compliance), will be evaluated using a paired-sample *t*-test, Wilcoxon rank-sum test, χ^2^ test, or Fisher’s exact test if the number of patients is matched between the NK and control groups. The position of lesions in the brain MRI will also be analyzed between the NK and control groups to determine the relationship between the position of infarction focuses and clinical manifestation of corneal damage. Related ocular indices will be analyzed between bilateral eyes. The incidence of different severities of corneal function will be calculated with the morbidity formula where incidence = number of NK patients/number of all enrolled patients. Crucially, a logistic regression model will be applied to the primary outcome between the subgroups. During the analysis, general and characteristic factors will be considered. All analyses will be considered statistically significant if the two-tailed P-value is < 0.05. Using univariate unconditional logistic regression, we will calculate the odds ratios and 95% confidence intervals for the association between clinical risk variables and NK. All analyses will be conducted using SPSS 20.0 software.

#### Missing data

The possibility of loss to follow-up and early deaths has been considered and will be calculated as a part of the sample size estimation. We strive to ensure the integrity of data. To reduce loss to follow-up, the staff at participating centers will reach out to participants on a regular basis, as needed in the follow-up period.

## Discussion

DLMI, also known as lateral medullary syndrome and posterior inferior cerebellar artery syndrome, is a cerebral infarction which has various manifestations; NK is a delayed-onset complication of DLMI [2, 3]. The lesions at the level of the spinal nucleus of the trigeminal nerve and spinal tract of the trigeminal nerve often cause NK [1, 2]. To date, there are no data on vascular conditions known to cause NK; few studies have investigated how DLMI influences corneal function, what happens to corneal innervation and the ocular surface environment after acute dorsolateral medullary infarction, and which susceptibility factors threaten the incidence and prognosis of NK.

As a keen peripheral afferent receptor, cornea provides different types of corneal neurons, such as polymodal nociceptors, neurons, cold thermoreceptor neurons, and selective mechanism-nociceptor neurons, which could maintain the sensation integrity of the cornea [3]. A mouse model was developed using trigeminal stereotactic electrolysis to destroy the ophthalmic branch of the trigeminal nerve; the study found an increase in cellular apoptosis and reduced proliferation in the epithelium [11]. Corneal sensation is attributed to the afferent receptor of the tear secretion loop [9]. Desiccation of the corneal surface and damage to the epithelial cells can aggravate the disease [3]; the imbalance of providing nutrition and removing cell waste persists, and ultimately results in corneal ulceration [9]. Thus, it is strongly suggested that early detection and interventions for NK should be conducted.

For the aforementioned reasons, we decided to conduct a unique, prospective observational study on dynamic histological corneal innervation and the ocular surface environment after DLMI from a clinical point of view. The ocular assessment is (semi) quantitively measured to reflect corneal innervation and the ocular surface environment. In this study, because IVCM allows for rapid, non-invasive, in vivo high-resolution imaging, it will be used to assess the corneal nerve [27]. Morphological alterations due to nerve impairment and repair will be reflected in the images. Visible parameters, such as nerve density, tortuosity, and thickness, are applicable because there will be a referenceable, standardized baseline [28]. The clinical significance of IVCM-based parameters will reflect relevance among morphological changes, corneal sensitivity, tear film production, and wound healing time. In addition, NK patients always have some ocular symptoms indicating an abnormal ocular surface, including dry eye and reduced blinking tear film instability, which are similar with those of dry eye disease (DED) [19]. A significant number of studies reported that structural and functional alterations of corneal nerves in patients with primary DED have neurosensory abnormalities and reduced corneal sensitivity [9]. Therefore, some representative examinations of DED will be used for NK patients because these two diseases have similar symptoms. Treatment of damaged ocular surface homeostasis in NK is the same as in DED, including relieving symptoms, preventing inflammation, tear substitution, and re-establishing a normal ocular surface [3, 9]. Furthermore, it is important to note that when the medullary reticular formation is involved, vasomotor instability and central hypoventilation can lead to cardiopulmonary arrest [22, 29]. Therefore, the issue will be kept in mind by each clinician and nurse as early as the first reception.

There are some limitations of the present study that must be addressed. First, since this registry focuses on patients with a rare disease, it may be difficult to recruit a sufficient number of patients for the study. Second, the sample may be less representative due to the exclusion of patients who are critically-ill and those who are unable to complete the established examinations; however, we do not think that these rare events will contribute to selection bias.

In conclusion, this study will be the first to observe the dynamic changes in corneal innervation and in the ocular surface environment after DLMI from a clinical point of view. Any manifestation after DLMI, such as diminished lacrimal secretions, diminished protective blink reflexes, photophobia, or impaired vision, should be considered by clinicians, and patients should strictly adhere to the treatment regimen suggested by their doctor. Otherwise, once a corneal transplant is required, there will not be enough time for doctors or patients to rescue the affected eye.

## Data Availability

This manuscript does not contain any data. We declare that the materials described in the manuscript will be freely available to any scientific group wishing to use them for reference for non-commercial purposes without breaching participant confidentiality. Data can be obtained from the corresponding author upon reasonable request, although confidential patient data will not be shared.

## Abbreviations

DLMI: Dorsolateralmedullary infarction
NK: Neurotrophickeratopathy
AIS: Acute ischemic stroke
OSDI: Ocular surfacedisease index
BSCVT: Best-spectacles corrected visual acuity
ASOCT: Anterior segmentoptical coherence tomography
IVCM: In vivo confocalmicroscopy
PBR: Partial blinking rate
NIHSS: National Institute ofHealth Stroke Scale
mRS: Modified Rankin scale
DED: Dry eye disease
